# Anti-Oxidative Effects of Human Adipose Stem Cell Conditioned Medium with Different Basal Medium during Mouse Embryo In Vitro Culture

**DOI:** 10.3390/ani10081414

**Published:** 2020-08-13

**Authors:** Kihae Ra, Hyun Ju Oh, Eun Young Kim, Sung Keun Kang, Jeong Chan Ra, Eui Hyun Kim, Byeong Chun Lee

**Affiliations:** 1 Department of Theriogenology and Biotechnology, College of Veterinary Medicine, Seoul National University, Seoul 08826, Korea; ragh1102@naver.com (K.R.); jooya5@snu.ac.kr (H.J.O.); hyun9214@daum.net (E.H.K.); 2 Biostar Stem Cell Research Institute, R Bio Co., Ltd., Seoul 08506, Korea; naraokke@stemcellbio.com (E.Y.K.); kangsk@stemcellbio.com (S.K.K.); jcra@stemcellbio.com (J.C.R.)

**Keywords:** adipose stem cell, anti-oxidative effect, basal medium, conditioned medium, in vitro culture, preimplantation embryo

## Abstract

**Simple Summary:**

Assisted reproductive techniques, which are used to resolve various infertility problems, have advanced following the emphasis on their use. Embryos produced in vitro rather than in vivo are exposed to greater stress, with the quality of the embryos being affected by the in vitro culture conditions. To reduce oxidative stress and consequent apoptosis of embryos for successful implantation and pregnancy maintenance, the present study evaluated the anti-oxidative effect of human adipose stem cell conditioned medium (ASC-CM) with different basal medium as supplement in in vitro culture (IVC) medium for mouse preimplantation embryo. Treatment of 5% human ASC-CM based on Dulbecco′s modified Eagle′s medium (DMEM-CM) indicated an enhanced development of mouse in vitro fertilized embryo, decreased expression level of indicators for oxidative stress, and apoptosis in blastocysts. To our knowledge, this is the first study to demonstrate that DMEM-CM can be an optimal supplement during IVC to promote in vitro embryo development and the success rate of assisted reproduction with its anti-oxidative and anti-apoptotic effects.

**Abstract:**

The quality of embryos produced by assisted reproductive techniques should be advanced by the improvement of in vitro culture conditions for successful implantation and pregnancy maintenance. We investigated the anti-oxidative effect of human adipose stem cell (ASC) conditioned medium with its optimal basal medium, Dulbecco′s modified Eagle′s medium (DMEM-CM), or keratinocyte serum-free medium (KSFM-CM) as supplements during in vitro culture (IVC) of in vitro fertilized mouse embryo. At first, preimplantation embryo development was evaluated in KSFM-CM and DMEM-CM supplemented cultures at various concentrations. The blastocyst (BL) and hatched BL formation rates were significantly increased in 5% DMEM-CM, while no difference was observed from KSFM-CM. Next, comparing the efficacy of KSFM-CM and DMEM-CM at the same concentration, DMEM-CM enhanced the developmental rate of 16 cells, morula, BL, and hatched BL. The expression level of reactive oxygen species decreased and that of glutathione increased in BL cultured with DMEM-CM, which confirms its anti-oxidative effect. Furthermore, apoptosis in BL cultured with DMEM-CM was reduced compared with that in KSFM-CM. This study demonstrated that the comparative effect of human ASC-CM made of two different basal media during mouse embryo IVC and anti-oxidative effect of 5% DMEM-CM was optimal to improve preimplantation embryo development.

## 1. Introduction

Infertility, defined as failure to become pregnant despite sexual intercourse without contraception for more than a year, is a prevalent condition among approximately 15% of couples worldwide [1]. To resolve the medical, social, and economic concerns that arise from infertility, assisted reproduction has been globalized, resulting in an increase in the birth rate globally [2]. According to the centers for disease control and prevention, the definition of assisted reproductive technologies (ARTs) covers all fertility treatments with the manipulation of gametes and embryos [3], and among ARTs, in vitro culture (IVC) is an essential step for the development of embryos, which can be implanted to result in successful pregnancy. Efforts have been made constantly to improve the IVC system for comparable quality and development of embryos in vivo [4]. However, although the exploration of the chemical composition of culture medium has upgraded the efficiency of assisted reproduction, the exact composition of reproductive tubal fluids still remains unrevealed [5]. Especially, the interaction of growth factors and cytokines from female reproductive tracts with embryos are required for improving the optimal microenvironment during IVC [6], and the supplementation of embryos in IVC medium with various growth factors has shown an effect to reduce apoptosis and degeneration, and increase blastocyst (BL) formation, total cell number, and hatching rate in various animal species [7].

Adipose stem cells (ASCs), a kind of mesenchymal stem cell, capable of self-renewal and embryonic lineage differentiation [8], have been widely applied to regenerative therapies with their competitive advantages. The strong proliferation ability of ASC is maintained regardless of the age of donors [9], and as compared with bone marrow stem cells, ASC can be easily and abundantly harvested from their source with a minimally invasive surgical procedure [10,11]. Notably, ASC secretes a variety of therapeutic cytokines and growth factors involved in apoptosis, immunomodulation, angiogenesis, and so on [12]. Antioxidation, mediated by secreted paracrine factors, is one of the most important effects of ASC as a regenerative medicine by inhibiting oxidative damage from reactive oxygen species (ROS) produced by environmental stress [13]. There have been studies on the relationship between ROS and reproduction including oocyte maturation, folliculogenesis, fertilization, and embryo development in vitro and in vivo [14]. The imbalance between ROS and antioxidants with ROS overproduction is associated with increased embryo fragmentation and apoptosis [15], decreased fertilization ability [16], and BL development [17], all of which lead to impaired embryo development and eventually failure of implantation [18]. As mentioned above, the secretory factors of ASC have shown anti-oxidative effects and the presence of these factors has been confirmed in conditioned medium (CM) [19], the medium containing the various factors produced during the source cell culture [20]. The application of CM has been evaluated and proven to possess comparable therapeutic capability as a substitute for original cell-based therapy [21]. In addition, CM has its own advantages in that the liquid formula with soluble factors is more convenient for use, storage, transportation, and mass-production, and is safer without immune rejection between cell donor and recipients [22]. However, the methods of manufacturing CM have not been standardized for culture duration, centrifugation conditions, and concentration protocol. Many types of basal media, for example, alpha minimum essential medium, Dulbecco’s modified Eagle’s medium (DMEM), and DMEM/F12, are currently used in CM, and the level of secreted growth factors in CM is known to be affected by culture medium [23]. A few studies have been conducted to compare keratinocyte serum-free medium (KSFM) with DMEM as cell culture medium [24,25,26], but to our knowledge, the difference between KSFM and DMEM as basal media of CM has never been investigated.

Prior to applying CM in human ARTs, it is required that the efficacy and toxicity of the materials are evaluated in animal models, allowing for insufficiency and ethical issues on human embryo [27,28]. Mouse preimplantation embryos are generally used to examine the development of culture medium owing to their developmental similarity to humans [29]. In the present study, using in vitro fertilized mouse embryos, we aimed to evaluate the anti-oxidative and anti-apoptotic effects of human ASC-CM with optimal basal medium by comparing KSFM and DMEM, and its ability as supplements to improve preimplantation embryo development during IVC.

## 2. Materials and Methods 

### 2.1. Ethics Approval and Consent to Participate

Human ASCs were provided from R Bio Stem Cell Research Center with the informed consent of cell donors to participate in the research and the approval by Life Ethics Committee of the Biostar Stem Cell Technology (RBIO 2015-12-001). The cells were tested prior to use in this study according to the Code of Federal Regulations, Title 21, Section 610.

### 2.2. Chemicals

All chemicals and reagents were purchased from Sigma-Aldrich (St. Louis, MO, USA), unless otherwise stated.

### 2.3. Human Adipose Stem Cell Culture and Characterization 

Human ASCs were isolated from the abdominal subcutaneous adipose tissues of three healthy female donors. The isolation and culture of human ASCs were performed as previously described [30]. Briefly, 1 × 10^6^ human ASCs, established and cryopreserved in advance, were thawed and seeded in a T-175 flask (175 cm^2^) with AMSC medium for adipose tissue-derived stem cell culture (R BIO, Seoul, Korea) in a humidified incubator at 37 °C and 5% CO_2_. The flasks of ASCs were cultured with complete culture medium for 4 days until confluent. The phenotypic characterization of cultured ASC was conducted using flow cytometry analysis. The ASCs were suspended in phosphate buffered saline (PBS) and 1 × 10^6^ of cells were labeled with fluorescein isothiocyanate (FITC) and phycoerythrin (PE), and then incubated with antibodies of mesenchymal stem cell (MSC) negative markers cluster of differentiation (CD) 31, CD34, and CD45, and positive markers CD73 and CD90 (BD Biosciences, San Jose, CA, USA) for 30–60 min. After washing with PBS, CD31-FITC, CD34-FITC, CD45-FITC, CD73-PE, and CD90-PE, markers were analyzed with a BD FACSCalibur™ flow cytometer and CellQuest Pro software (BD Biosciences, San Jose, CA, USA).

### 2.4. Preparation of Adipose Stem Cell Conditioned Medium

To collect CM of different basal media, two flasks of ASCs per donor were cultured with AMSC medium for adipose tissue-derived stem cell culture (R BIO, Seoul, Korea) for 2 days and then the medium of each flask was changed with defined KSFM or DMEM after washing twice with PBS. The KSFM and DMEM were collected after 24 h and fresh KSFM and DMEM were added respectively to the original flask. After repeated supernatant collection for 5 days, KSFM- and DMEM-CM harvested on different dates were pooled, centrifuged at 2500 rpm for 5 min, and filtered using a 0.22 µm filter. KSFM- and DMEM-CM from three donors were then mixed in a total volume of 20 mL and centrifuged at 3000× *g* for 90 min at 4 °C using a 3 kDa cut-off filter tube (Vivaspin 20; GE healthcare, Chicago, IL, USA) until concentrated to the final volume of 2 mL. The composition of DMEM is described in Table 1, whereas the formulation of KSFM is undisclosed by the manufacturer.

### 2.5. Experimental Animals

Seven- or eight-week-old female and 10- or 11-week-old male ICR mice were purchased from Orient Bio (Gapyeong, Korea) and housed under conventional conditions in an animal facility in Seoul National University. All experimental animals used in this study were kept in a room with a 12 h light/12 h dark cycle and controlled temperature and humidity (23 °C, 60%). The experimental protocols were approved by the Institutional Animal Care and Use Committee of Seoul National University (SNU-170511-2-4) in accordance with the principles of the 3Rs (replacement, reduction, and refinement).

### 2.6. In Vitro Fertilization

The procedures for in vitro fertilization were all conducted according to the manual of Center for Animal Resources and Development (CARD) on reproductive engineering techniques in mice [31]. First, caudal epididymides were removed from mature male mice after cervical dislocation and placed in a petri dish with a droplet of 90 µL CARD medium (CosmoBio, Tokyo, Japan) covered with mineral oil. The duct of the caudal epididymis was incised using a 29-gauge needle and sperm stored inside was dispersed into the droplet. The sperm was incubated at 36 °C for an hour for capacitation. To collect oocytes, superovulation was induced by intra-peritoneal injection of 10 IU pregnant mare serum gonadotropin and 10 IU human chorionic gonadotropin (hCG) followed 47–48 h later. Mature female mice were euthanized by cervical dislocation 15–17 h after hCG administration. The cumulus-oocyte complexes (COCs) were recovered from the oviductal ampulla and moved to a droplet of 200 µL CARD medium. For insemination, 10 µL of sperm suspension was introduced to the droplet where COCs were placed and incubated for 3 h. Inseminated oocytes were washed in human tubal fluid (HTF; CosmoBio) at least three times and cultured in a fresh droplet of HTF (day 1). After 24 h, the morphology of embryos was assessed under a stereomicroscope. Unfertilized and fragmented embryos were removed and only embryos that developed to the two-cell or four-cell stage were randomly divided into experimental groups for IVC in humidified incubator at 36 °C with 5% O_2_ and CO_2_ (day 2).

### 2.7. Experimental Groups

For the determination of optimal concentration for KSFM- and DMEM-CM treatment during IVC, in vitro fertilized embryos (*n*) were cultured in continuous single culture-NX (CSCM-NX; FUJIFILM Irvine Scientific, Santa Ana, CA, USA) medium containing 2.5% and 5% (*v*/*v*) KSFM-CM treated groups and a control group consisting of 100% CSCM-NX from days 2 to 5 (*n* = 270), and DMEM-CM were tested with the same method (*n* = 208). According to the blastocyst formation rate assessed on day 5, the respective concentration for KSFM- and DMEM-CM treatment was decided and, finally, the KSFM- and DMEM-CM treated groups were compared (*n* = 268). Six female and one male mice were used for each in vitro fertilization, which was replicated six times in total. The composition of CSCM-NX is listed in Table 2.

### 2.8. Embryo Development Evaluation and Total Cell Counts

On days 4 and 5, the developmental stages of embryos in the control and treatment groups were evaluated, including 4-cell, 8-cell, 16-cell, and BL, and the hatching rate of BL was assessed on day 5 using a stereomicroscope. The total cell number of BL was counted on day 5 after staining with 5 µg/mL Hoechst 33,342 for 12 min. The BLs were washed in PBS after staining, moved to glycerol drops on a glass slide, and then covered with a glass coverslip. The total number of cells in BLs was observed under a fluorescence microscope (Nikon Corp., Tokyo, Japan).

### 2.9. Measurement of ROS and Glutathione (GSH) Levels

For the measurement of intracellular ROS and GSH levels, BLs from each group were stained with H2DCFDA (2′,7’-dichlorodihydrofluorescein diacetate) (*n* = 30) and CellTracker Blue (4-chloromethyl-6,8-difluoro-7-hydroxycoumarin; CMF2HC) (*n* = 30), respectively, on day 5. The BLs were washed and incubated for 30 min in 1% PBS containing polyvinyl alcohol (PVA-PBS) diluted with 10 μM H2DCFDA or CellTracker Blue at 23 °C in the dark. BLs were transferred to a 4 μL droplet of PVA-PBS covered with mineral oil and then the fluorescence intensity was measured using an epifluorescence microscope (TE2000-S; Nikon, Tokyo, Japan) with UV filters (460 nm for ROS and 370 nm for GSH). The analysis of fluorescence intensity was performed using Image J software version 1.52 (National Institutes of Health, Bethesda, MO, USA).

### 2.10. Immunofluorescence Staining

The expression levels of cleaved caspase 3 were measured using indirect immunofluorescence staining in BL from each group (*n* = 45). The BLs were collected on day 5, washed in 1% PVA-PBS, and then fixed with 4% paraformaldehyde-PBS for 1 h. For permeabilization, BLs were washed in 1% PVA-PBS three times and incubated at 36 °C in 1% Triton X-100 in 1% PVA-PBS. After 1 h, BLs were washed in 1% PVA-PBS five times and incubated at 36 °C in 2% bovine serum albumin-PBS. The BLs were incubated with cleaved caspase-3 primary antibody (#9661; Cell Signaling, Boston, MA, USA) diluted with 2% BSA-PBS in 1:400 at 4 °C overnight, and then incubated with goat anti-rabbit fluorescein isothiocyanate conjugated secondary antibody in 2% BSA-PBS in 1:400 at 36 °C for 2 h after being washed in 1% PVA-PBS three times. As negative control, BLs were incubated with secondary antibody, while primary antibody was omitted. BLs were washed in 1% PVA-PBS and then counterstained with 5 µg/mL Hoechst 33,342 for 12 min, followed by mounting on glycerol drops on a glass slide covered with a glass coverslip. An epifluorescence microscope was used to measure the fluorescence intensity of cleaved caspase-3, which was quantified using the Image J software version 1.52.

### 2.11. Statistical Analysis

All experiments were repeated at least three times and statistical analyses were performed using the Mann–Whitney test and the GraphPad Prism software version 5 (GraphPad, San Diego, CA, USA). Data are presented as means ± standard error of mean. *p*-value less than 0.05 among groups was considered as statistically significant.

## 3. Results

### 3.1. Characterization of Isolated ASCs

**Figure 1:**
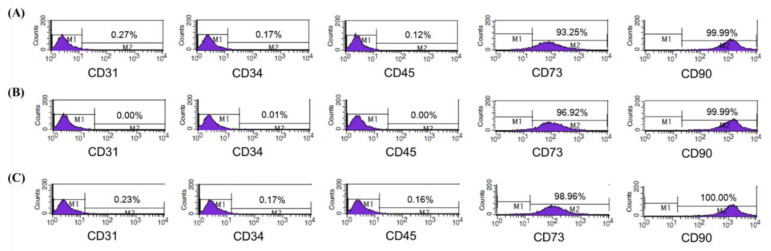
Phenotypic characterization of adipose-derived stem cell (ASC). Flow cytometric analysis of ASCs isolated from three female donors (**A**–**C**). Cluster of differentiation (CD) 73, CD90: mesenchymal stem cell specific marker. CD31: endothelial cell specific marker. CD45: hematopoietic lineage marker. CD34: hematopoietic stem cell specific marker.

Flow cytometric analysis was conducted to identify the expression of mesenchymal stem cell (MSC)-specific markers in isolated ASCs from healthy female donors (Figure 1A–C). ASCs from all donors expressed CD73 and CD90, typical MSC surface markers, but not CD31, CD34, or CD45, known as markers of endothelial cell, hematopoietic stem cell, and hematopoietic lineage, respectively. As a result of phenotypic marker assessment, the isolated cells used for CM were proven as ASCs and qualified for the subsequent experiments.

### 3.2. Effects of KSFM-CM on the Developmental Competence of Embryo

**Figure 2:**
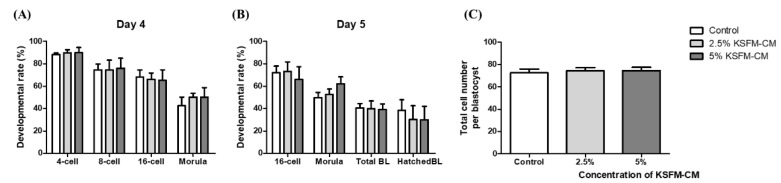
Effects of 2.5% and 5% keratinocyte serum-free conditioned medium (KSFM-CM) on the development of in vitro fertilized embryo. The rate of embryos developed to each stage was assessed on (**A**) day 4 and (**B**) day 5 from in vitro culture (IVC) with KSFM-CM; (**C**) total cell number of BL on day 5 was counted by Hoechst staining. Data are expressed as the mean ± standard error of the mean (SEM).

To determine the optimal concentration of KSFM-CM supplementation, in vitro fertilized embryos were divided and cultured in control and treatment groups with 2.5 and 5% KSFM-CM. The rate of development of embryos of each group was assessed on days 4 and 5. From the result of in vitro development in 0, 2.5, and 5% KSFM-CM, there was no significant difference in the rate of embryos developed to 4-, 8-, and 16-cell; morula stage; BL formation rate; and hatched BL formation rate among groups on day 4 (Figure 2A) and day 5 (Figure 2B). Total cell number of BL developed from each group on day 5 was counted and no significant difference was observed among all groups (Figure 2C).

### 3.3. Effects of DMEM-CM on Developmental Competence of Embryo

**Figure 3:**
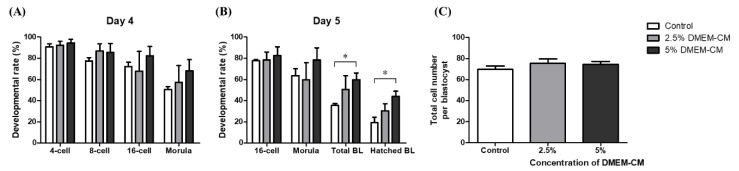
Effects of 2.5% and 5% Dulbecco′s modified Eagle′s medium (DMEM-CM) on the development of in vitro fertilized embryo. The rate of embryos developed to each stage was assessed on (**A**) day 4 and (**B**) day 5 from IVC with KSFM-CM; (**C**) total cell number of BL on day 5 was counted by Hoechst staining. Data are expressed as the mean ± standard error of the mean (SEM). * *p* < 0.05.

The effect of 0, 2.5, and 5% DMEM-CM treatment was evaluated using the same method as used for KSFM-CM. As shown in Figure 3A, no significant difference was observed in 4-, 8-, and 16-cell and morula stage embryo development among groups on day 4. However, the formation rate of both BL and hatched BL in the 5% DMEM-CM group was significantly higher than those of the control group (59.83 ± 6.37 vs. 35.58 ± 1.81%, 44.05 ± 4.90 vs. 19.23 ± 5.22, *p* < 0.01), while there was no difference between 2.5% DMEM-CM and the other groups on day 5 (Figure 3B). With respect to total cell numbers of BL, there was no significant difference among groups (Figure 3C).

### 3.4. Comparative Effects of KSFM-CM and DMEM-CM on Embryo Development

**Figure 4:**
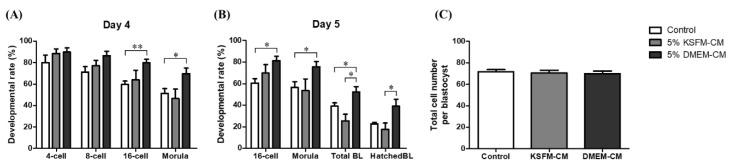
Comparative effects of KSFM-CM and DMEM-CM on the development of in vitro fertilized embryo. The rate of embryos developed to each stage was assessed on (**A**) day 4 and (**B**) day 5 from IVC with 5% KSFM-CM and 5% DMEM-CM. (**C**) Total cell number of BL on day 5 was counted by Hoechst staining. Data are expressed as the mean ± standard error of the mean (SEM). * *p* < 0.05, ** *p* < 0.01.

On the basis of previous experiments, the concentration of 5% was determined for comparing KSFM-CM and DMEM-CM. The developmental rate of 16-cell stage embryos (79.96 ± 3.21 vs. 59.64 ± 3.11, *p* < 0.01) and morula (69.80 ± 5.09 vs. 51.40 ± 4.43, *p* < 0.05) in 5% DMEM-CM group was significantly higher than that of the control group, but not higher than the 5% KSFM-CM group on day 4 (Figure 4A). Significant difference was observed in the developmental rate of 16-cell (60.58 ± 4.06 vs. 81.26 ± 4.30, *p* < 0.05) and morula (56.48 ± 5.41 vs. 75.62 ± 4.92, *p* < 0.05) between the control and the 5% DMEM-CM group on day 5. BL formation rate in the 5% DMEM-CM group (52.32 ± 4.88) increased significantly to that in the control (39.00 ± 3.21, *p* < 0.05) and 5% KSFM-CM groups (25.54 ± 6.11, *p* < 0.05) on day 5. Lastly, the formation rate of hatched BL in the 5% DMEM-CM group (39.04 ± 6.61) was significantly higher than that in the 5% KSFM-CM group (17.50 ± 5.99, *p* < 0.05), but no difference was observed in the control group on day 5 (Figure 4B). There was no difference in total cell number of BL among the groups (Figure 4C).

### 3.5. Effects of KSFM-CM and DMEM-CM on Oxidative Stress in BL

**Figure 5:**
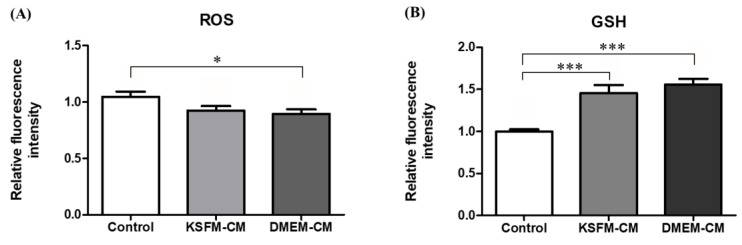
Quantification of reactive oxygen species (ROS) and glutathione (GSH) levels in blastocyst (BL) culture with 5% KSFM-CM and 5% DMEM-CM. Fluorescence intensity of (**A**) ROS and (**B**) GSH was measured in BL on day 5. Data are expressed as the mean ± standard error of the mean (SEM). * *p* < 0.05, *** *p* < 0.001.

Intracellular levels of ROS and glutathione (GSH) in BL from the control, 5% KSFM-CM, and 5% DMEM-CM groups collected on day 5 were measured to assess the anti-oxidative effects of ASC-CM. As indicated in Figure 5A, ROS levels of BL in the 5% DMEM-CM group were significantly lower than in the control group (*p* < 0.05), but the 5% KSFM-CM group showed similar expression in the control and 5% DMEM-CM groups. The BL cultured in both 5% DMEM-CM and KSFM-CM increased significantly in GSH level compared with the control (*p* < 0.001, Figure 5B).

### 3.6. Effects of KSFM-CM and DMEM-CM on Apoptosis in BL

**Figure 6:**
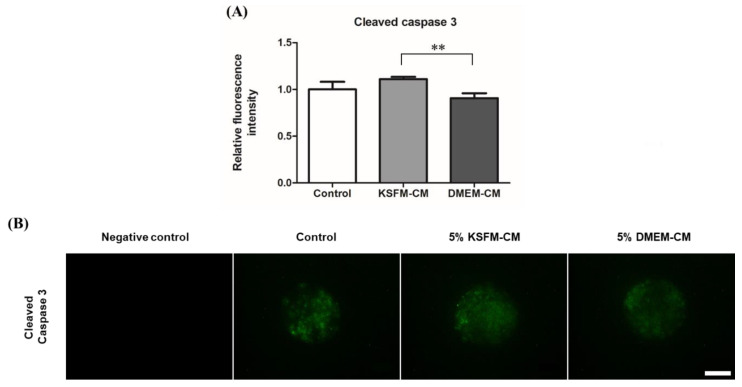
Detection of cleaved caspase 3 in BL cultured with 5% KSFM-CM and 5% DMEM-CM. (**A**) Fluorescence intensity of cleaved caspase 3 was quantified in BL on day 5 by immunofluorescence staining. Data are expressed as the mean ± standard error of the mean (SEM). ** *p* < 0.01. (**B**) Representative immunofluorescent images for cleaved caspase 3 (green) in BL. Original magnification 400×. Scale bar = 50 μm.

Cleaved caspase-3, the active form of apoptosis executioner, present in apoptotic cells of BL, was visualized using immunocytochemistry (Figure 6B) and the fluorescence intensity of each group was evaluated. No fluorescence was detected in negative control. The level of cleaved caspase 3 decreased significantly in the 5% DMEM-CM group compared with that in the 5% KSFM-CM group, but was similar to that in the control (*p* < 0.01, Figure 6A).

## 4. Discussion

To our knowledge, this is the first study to investigate the effect of human ASC-CM, made of two different basal media, DMEM and KSFM, on mouse in vitro fertilized embryo development as supplemented with IVC media at an optimal concentration. The indicators of oxidative stress and apoptosis, ROS, GSH, and cleaved caspase-3 were assessed in BL developed from the DMEM-CM and KSFM-CM groups. Our results demonstrated that 5% DMEM-CM improved preimplantation embryo development with its anti-oxidative and anti-apoptotic effects compared with the same concentration of KSFM-CM.

The beneficial effects of IVC with ASC or ASC-CM on embryo development have been reported in recent studies [32]. According to a previous study, a variety of growth factors were found in human ASC-CM including fibroblast, transforming, vascular endothelial, epidermal, insulin-like, granulocyte-macrophage colony-stimulating, and platelet-derived growth factors [19,33]. These are representative factors with supportive effects on preimplantation embryo development, which have been demonstrated for decades, in addition to an increase in blastocyst formation rate, cell number, expansion, hatching, protein synthesis, and decrease in apoptosis [7,34,35,36,37,38]. Although the effectiveness of these factors present in ASC-CM has been proven, the treatment concentration should be determined in order to develop an optimal IVC medium. A high concentration of CM should not be conclusively selected without considering that increasing the quantity of cytokines does not ensure advanced capability because both stimulatory and inhibitory factors can be present in CM [39]. Indeed, the growth rate of human endometrial microvascular endothelial cells in the 20% human embryo CM treated group was reduced in contrast to the 2.5 and 10% treated groups, and cell proliferation was stimulated the most in the concentration of 5% [40]. Regarding human dermal fibroblasts under cytotoxic condition, 2.5 and 5% CM of human natural killer cell enhanced cell proliferation 72 h after treatment, while the 10% treatment group showed no significant effect [41]. Notably, in our previous experiment, the effect of 25% DMEM-CM on blastocyst formation rate in parthenogenetically activated mouse embryos when treated during IVC was detrimental, but 10% DMEM-CM indicated no difference compared with the control group (data not shown). In the present study, 5% DMEM-CM significantly enhanced the rate of blastocyst and hatched blastocyst development from two-cell embryos on day 5 compared with that of the control group (Figure 3A,B), which was finally chosen as the concentration of treatment in subsequent experiments.

We then focused on the comparison of KSFM-CM and DMEM-CM treatment at 5% concentration, in order to select a more suitable basal medium to be applied as embryo IVC medium supplements. Among the embryos that developed to more than 16 cell stages, morula is characterized by its appearance with unclear outlines of blastomeres following the process of compaction, and observed from day 3 or 4 after fertilization [42]. In fact, embryonic genome activation is known to generally occur in a part of morula stage, which causes embryos that undergo this phase to possibly proceed to compaction and cavitation [43]. Transcribed genes in this stage, engaged in metabolism of amino acids, carbohydrates, lipids, and proteins, regulate the system of energy metabolism in embryos, which is required for consequent development into BL [44]. In the aspect of clinical embryology, the morphological status of morula can be associated with subsequent quality and rate of development of BL; moreover, embryo transfer of morula on day 4 of proposed pregnancy, implantation, and live birth rate was comparable to that of blastocyst on day 5 [45]. From the results, the DMEM-CM group showed a significantly higher incidence of embryo development to the 16-cell stage and morula than in the control, and development to morula stage was significantly improved compared with that of the KSFM-CM group on both days 4 and 5 (Figure 4A,B).

We continued to monitor whether morula, observed on day 4, continuously developed to BL on day 5. The production of viable BL cultured and developed in vitro is important because the interaction between BL and the uterine endometrium determines the success of implantation [46]. The advanced capability of BL, especially zona pellucida hatching, is a critical precondition for high implantation and early pregnancy rates [47], considering that hatching failure can partially induce relatively low implantation rates in the process of ARTs [48]. Our results indicated that DMEM-CM significantly enhanced total BL formation rate on day 5 compared with the other groups and the rate of hatched BL improved significantly in DMEM-CM compared with that of KSFM-CM (Figure 4A,B).

Next, we analyzed the expression of ROS, GSH, and cleaved caspase 3 in BL obtained from IVC with KSFM-CM and DMEM-CM. Successful implantation and pregnancy are affected by not only the number, but also the quality of transferred BL [49]. The quality of in vitro produced BL is positively or negatively associated with its intracellular ROS levels [50]. Previous studies have demonstrated that a moderate amount of ROS is required in reproductive functions and an appropriate level of increase may be observed in certain processes [51]. The rise in ROS has been reported at the embryo compaction and blastocyst stage, presumably caused by the transition of the ATP production system from anaerobic to aerobic glycolysis [52], and at hatching programmed to protect embryos from a drastic change in superoxide [53]. However, oxidative stress occurs as a result of the imbalance between disproportionate levels of ROS and antioxidants, which can regulate the associated disorders [54]. An excessive amount of ROS can be generated in in vitro produced embryos by exogenous factors at a suboptimal microenvironment or improper manipulations during ARTs [55]. GSH, one of the most abundant and representative antioxidants synthesized and present in cells [56], mainly protects cellular functions from oxidative damage and subsequent apoptosis [57]. In this study, DMEM-CM and KSFM-CM significantly increased intracellular expression of GSH, and DMEM-CM decreased ROS level in BL compared with those of the control group (Figure 6A,B), in accordance with our aim to reduce oxidative stress of embryos during IVC with the anti-oxidative effects of human ASC-CM, as previously described [19].

Lastly, in this study, we showed the anti-apoptotic effect of DMEM-CM from which cleaved caspase 3 was expressed less in BL from the DMEM-CM group than the KSFM-CM group. Apoptosis is one of the evaluation indexes for the quality of BL [58], which can be improved by a decrease in cell death [59]. Caspase 3 in pro-apoptotic caspase subfamilies modulates cell death signal and executes apoptosis once activated and cleaved [60]. Cleaved caspase 3 can be identified with its specific epitope expressed only after caspase 3, present as procaspase in normal state, activated by apoptotic pathways [61], and thus immunodetection of cleaved caspase 3 has generally been applied for measuring apoptosis [62]. Suboptimal culture condition with elevated levels of ROS can discourage preimplantation embryo development [17] and increase cellular apoptosis of BL [63], leading to an unexpected outcome of assisted reproduction [64]. Our results suggest the apoptosis of BL was inhibited with DMEM-CM treatment, which had a positive effect on the environment of embryo culture compared with KSFM-CM.

## 5. Conclusions

The present study demonstrated that DMEM-CM can be an optimal supplement in culture medium, promoting in vitro embryo development and the success rate of assisted reproduction with its anti-oxidative and anti-apoptotic effects in comparison with KSFM-CM. On the basis of this study, we expect to establish novel culture media for human preimplantation embryo using DMEM-CM, but further studies are required to elucidate their undiscovered effects.

## Figures and Tables

**Table 1:** The composition of Dulbecco’s Modified Eagle Medium (DMEM) | Sigma-Aldrich D6429.

DMEM		
Component		(g/L)
Inorganic Salts	CaCl_2_	0.2
	Fe(NO_3_)_3_ • 9H_2_O	0.0001
	MgSO_4_	0.09767
	KCl	0.4
	NaHCO_3_	3.7
	NaCl	6.4
	NaH_2_PO_4_	0.109
Amino Acids	L-Arginine • HCl	0.084
	L-Cystine • 2HCl	0.0626
	L-Glutamine	0.584
	Glycine	0.03
	L-Histidine • HCl • H_2_O	0.042
	L-Isoleucine	0.105
	L-Leucine	0.105
	L-Lysine • HCl	0.146
	L-Methionine	0.03
	L-Phenylalanine	0.066
	L-Serine	0.042
	L-Threonine	0.095
	L-Tryptophan	0.016
	L-Tyrosine • 2Na • 2H_2_O	0.10379
	L-Valine	0.094
Vitamins	Choline Chloride	0.004
	Folic Acid	0.004
	myo-Inositol	0.0072
	Niacinamide	0.004
	D-Pantothenic Acid • ½Ca	0.004
	Pyridoxine • HCl	0.00404
	Riboflavin	0.0004
	Thiamine • HCl	0.004
Other	D-Glucose	4.5
	Phenol Red • Na	0.0159
	Pyruvic Acid • Na	0.11

**Table 2:** The composition of continuous single culture (CSCM)-NX | Irvine Scientific.

	CSCM-NX
Inorganic Salts	Calcium Chloride
	Magnesium Sulfate
	Potassium Chloride
	Potassium Phosphate
	Sodium Chloride
Amino Acids	Alanine
	Arginine
	Asparagin
	Aspartic acid
	Cystine
	Glutamic acid
	Glycine
	Histidine
	Isoleucine
	Leucine
	Lysine
	Methionine
	Phenylalanine
	Proline
	Serine
	Threonine
	Tryptophan
	Tyrosine
	Valine
Energy Substrates	Dextrose
	Sodium Lactate
	Sodium Pyruvate
Antioxidants	EDTA
	Sodium Citrate
Dipeptide	Alanyl-glutamine
Buffer	Sodium Bicarbonate
Antibiotic	Gentamicin
